# Novel Multimodal, Multiscale Imaging System with Augmented Reality

**DOI:** 10.3390/diagnostics11030441

**Published:** 2021-03-04

**Authors:** Christopher Mela, Francis Papay, Yang Liu

**Affiliations:** 1 Iowa Technology Institute, The University of Iowa, 103 South Capitol Street, Iowa City, IA 52242, USA; cam189@zips.uakron.edu; 2 Department of Biomedical Engineering, The University of Akron, 302 E Buchtel Ave, Akron, OH 44325, USA; 3 Dermatology & Plastic Surgery Institute, Cleveland Clinic, Cleveland, OH 44195, USA; papayf@ccf.org; 4 Department of Electrical and Computer Engineering, The University of Iowa, 103 South Capitol Street, Iowa City, IA 52242, USA; 5 Iowa Institute for Biomedical Imaging, The University of Iowa, 169 Newton Road, Iowa City, IA 52242, USA; 6 Center for Bioinformatics and Computational Biology, The University of Iowa, 103 South Capitol Street, Iowa City, IA 52242, USA; 7 Iowa Informatics Initiative, The University of Iowa, 145 N Riverside, Iowa City, IA 52242, USA

**Keywords:** multimodal imaging, multiscale imaging, computer vision, augmented reality, fluorescence imaging

## Abstract

A novel multimodal, multiscale imaging system with augmented reality capability were developed and characterized. The system offers 3D color reflectance imaging, 3D fluorescence imaging, and augmented reality in real time. Multiscale fluorescence imaging was enabled by developing and integrating an in vivo fiber-optic microscope. Real-time ultrasound-fluorescence multimodal imaging used optically tracked fiducial markers for registration. Tomographical data are also incorporated using optically tracked fiducial markers for registration. Furthermore, we characterized system performance and registration accuracy in a benchtop setting. The multiscale fluorescence imaging facilitated assessing the functional status of tissues, extending the minimal resolution of fluorescence imaging to ~17.5 µm. The system achieved a mean of Target Registration error of less than 2 mm for registering fluorescence images to ultrasound images and MRI-based 3D model, which is within clinically acceptable range. The low latency and high frame rate of the prototype system has shown the promise of applying the reported techniques in clinically relevant settings in the future.

## 1. Introduction

Limitations on the operational functionality of single-mode medical imaging technologies in guided interventions necessitate the inclusion of multiple regimes to attain a fuller measure of the field lain before. Many intraoperative medical imaging modalities have gained more prominent use in the surgical suite in the past 20 years. Fluorescence imaging methods have seen a particular increase in research and clinical support ever since the release of the impactful Novadaq SPY in 2006 [1], and later the publication of the FLARE imaging system, released in 2009 [2]. The ability to actively or passively target, label, and enhance the visibility of blood vessels, lymph nodes, and cancerous or pre-cancerous lesions beneath the tissue surface has found use in many operative procedures and surgical planning [3,4,5]. Fluorescence imaging has received significant interest from researchers in the last decade [6,7,8,9,10,11,12,13]. A limitation of fluorescence imaging is the inability to provide significant contrast support at tissue depths beyond a few millimeters from the surface. The development and expansion of near-infrared (NIR) fluorescent dye has extended this range to as much as 1–2 cm [14,15]; however, alternate modalities are still required for comprehensive volumetric imaging. The tradeoff between the field of view and resolution also calls for multiscale fluorescence imaging for comprehensive functional assessment.

Tomographical techniques such as magnetic resonance imaging (MRI) and X-ray computed tomography (CT) provide useful structural and volumetric data at any depth through the living tissue, although they lack the fine resolution of fluorescence and the convenience of ultrasound for real-time imaging [3,16,17]. Ultrasound has proven to be a ubiquitous tool in many medical settings, providing quick anatomical readings of soft tissue at depths of several centimeters [18,19,20]. Attaining fine resolution in ultrasound images can be difficult, and it may not be easy for the uninitiated to orient the transducer correctly. Multiple studies have been conducted to merge two or more of the aforementioned techniques, leveraging one’s strengths to offset the weaknesses of the other.

Each imaging method has its limits. Fluorescence imaging provides excellent fine resolution and selective targeting; however, it suffers from low tissue depth penetration [21,22,23,24]. Tomographical techniques, such as MRI and CT, have good volumetric resolution but lack the same level of accessibility as ultrasound and the sensitivity of fluorescence imaging [25]. Radiological methods such as PET can aid in selectively distinguishing a pathological target within a tissue volume; however, it lacks spatial resolution when used independently. Various methods, including hardware and software algorithms, have been implemented to facilitate the registration of different imaging modalities.

Registration of image modalities may involve the use of fiducials or markers. Recognition of a unique physical marker through its optically distinct pattern, infrared emission profile, or electromagnetic signature allows researchers to designate an image registration site. Typically, there are two methods of marker-based registration, optical-based and virtual-based. Virtual registration tracks and monitors the registration markers’ real-world locations and translates those locations into virtual space, where the registered image data can be displayed. Optical-based methods may operate similar to the virtual methods; however, they incorporate optical imaging, registering the tracked image data onto the scene recorded by a video camera.

A variety of fiducial markers have been implemented for surgical planning or intraoperative guidance. Infrared (IR) markers that attach to a target, such as patient anatomy or medical instrument, have become a popular option, not requiring optical imaging methods for implementation. Two main categories of IR markers have found regular use, those that emit IR light and those that reflect IR light. Markers are tracked by a separate IR-sensitive instrument, such as the NDI Optotrak [26], and must remain within the device’s field-of-view (FOV) for continuous tracking. Both markers have found use with ultrasound probe tracking [27,28,29,30,31,32] and the registration of tomographical imagery [33,34,35].

Optically tracked markers use a distinctive shape or pattern for computer vision-based recognition. These markers are typically inexpensive and non-complicated; however, they must remain in the camera’s field-of-view (FOV). Additionally, optically tracked markers typically do not rival IR probes or electromagnetic (EM) trackers for accuracy. Printed patterned markers, such as those incorporated into the Aruco system, provide a robust and very inexpensive solution [36]. Similar methods use other distinctive patterns such as chessboards [37,38,39,40], dot arrays [31,41,42], or matrix barcodes [43]. Patterned markers have been used for ultrasound tracking for co-registration with video navigation [32,44,45], and tomographical [40,46,47] data co-registration. Certain optically tracked registration regimes require the use of fiducials that are visible to multiple imaging modalities. Methods include non-magnetic metal or metallic-filled markers for MRI or CT registration [48,49,50,51] with optical modalities or between preoperative and intraoperative images, as well as ultrasound sensitive fiducials.

While typically not as accurate as IR tracking methods, EM markers still provide a high degree of acuity without the requirement of FOV imaging. However, EM marker locations must still be localized with respect to the image FOV during optical imaging, which is not a trivial task, particularly, when the marker is not located within the imaging frame FOV. Nonetheless, EM has proven to be an effective option for medical imaging co-registration, both with ultrasound [52,53,54,55,56] as well as tomographic image [57,58] representation.

This study developed and characterized a novel multimodal, multiscale imaging system with augmented reality capability. The system offered 3D color reflectance imaging, 3D fluorescence imaging, and augmented reality (AR) in real time. Multiscale fluorescence imaging was enabled by developing and integrating an in vivo fiber-optic microscope. Real-time ultrasound-fluorescence multimodal imaging used optically tracked fiducial markers for registration. Tomographical data were also incorporated using optically tracked fiducial markers for registration. Furthermore, we characterized system performance regarding operational frame rate and lag, the accuracy of fiducial tracking and multimodal image registration, and microscopic resolution and detection sensitivity.

## 2. Materials and Methods

### 2.1. Multimodal Optical Imaging and Display

**Figure 1:**
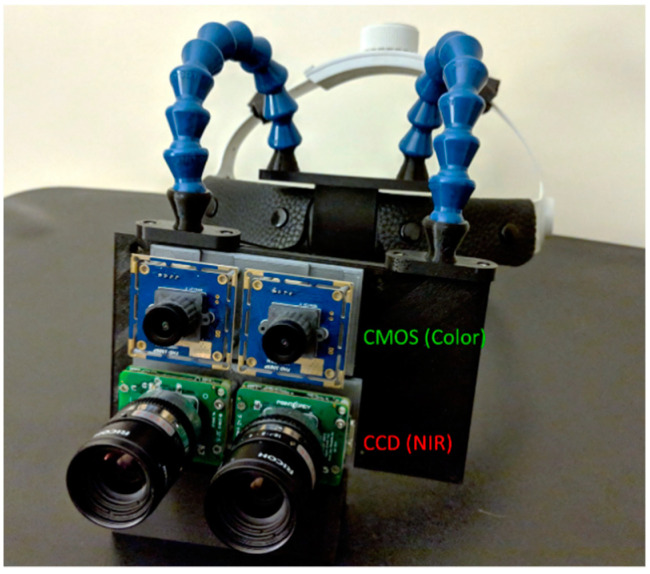
Head-mounted stereoscopic multimodal 3D optical imaging AR display system. Two CCD cameras are used for near-infrared (NIR) fluorescence imaging and two CMOS cameras are used for color reflectance imaging. The imaging module is connected to a loupe-style head mount.

Stereoscopic fluorescence imaging was conducted using twin monochrome CCD imaging sensors (CM3-U3–13S2M-CS FLIR, Richmond, BC, Canada). Color reflectance mode imaging was conducted using two CMOS cameras (USBFDH01M ELP, Shenzhen, China). The CCD fluorescence imaging sensors were fitted with 832 nm bandpass filters to optimize the system for indocyanine green (ICG) detection, while the CMOS sensors were equipped with NIR cutoff filters, to prevent IR contamination of the color imagery (84-091 and 84-107 Edmund Optics, NJ, USA). Lensing included 8 mm focal length C-mount lenses (M118FM08 Tamron, Saitama, Japan) for the CCD cameras and glass 8 mm M12 lenses for the CMOS sensors. Sensors were mounted onto a custom 3D-printed housing, Figure 1, aligning each like pair of sensors together horizontally, and aligning the CCD sensors vertically over the CMOS. A stereoscopic VR display was attached to the back of the camera housing via a secured clip. The assembled imaging module was affixed to a head mount using articulating arm connectors.

Fluorescence excitation of the NIR dye ICG (I2633 Sigma Aldrich, St. Louis, MO, USA) was induced using an adjustable focus, heatsink-mounted 780 nm LED emitter with an 800 nm short pass filter (M780LP1, SM1F and FES0800 ThorLabs, Newton, NJ, USA). White light reflectance mode illumination was provided using a white light LED emitter with independently adjustable optics and 800 nm short pass filter (MNWHL4 and FES0780 ThorLabs, Newton, NJ, USA). Both white light and NIR emitters were connected to separate, independently adjustable LED driver circuits (LEDD1B ThorLabs, Newton, NJ, USA).

### 2.2. Ultrasound Imaging

The ultrasound imaging system (Ultrasonix, Shenzhen, China) was integrated into the computational unit via Data Acquisition Card (DAC) (USB-Live2 Hauppauge, Suffolk County, NY, USA) and USB connection. The system utilized a 3.5 MHz convex probe and ultrasound transmission gel (Aquasonic 100 Parker, Washington Township, NJ, USA) applied topically to the target surface to enhance sonic transduction and resultant image clarity.

### 2.3. Computation

The imaging and display modules were connected to a mini-PC (NUC6i7KYK Intel, San Francisco, CA, USA), which controlled the cameras, captured and processed input imaging frames from both the ultrasound and camera modules, integrated multimodal registration via optically tracked fiducial markers and output display frames to the head mounted AR unit as well as to a stand-alone monitor. The system was equipped with 32GB 2133 MHz DDR4 Ram and a 500 GB SSD, while the CPU operated an Intel^®^ Core™ i7-6770HQ processor at 2.6 MHz per core with Intel^®^ Iris™ Pro 580 integrated graphic card. Camera and ultrasound DAC connections were made using USB 3.0 ports, while the AR display was connected via HDMI.

### 2.4. Ultrasound-to-Fluorescence Registration

Ultrasound images were registered directly to the transducer using Aruco markers as fiducial points. The Aruco markers were mounted onto a 3D-printed housing before being affixed onto the transducer, similar to [45]. The 3D-printed housing was topped with a diffuse plastic pane on which the markers sat. Inside the box were placed a set of white and 830 nm LEDs, along with batteries. When one set of LEDs were switched on, the markers were illuminated from below, improving marker visibility by either the color or fluorescence imaging cameras. The LEDs were driven by an RC circuit with steady 3V source, whose parameters were calibrated through trial and error to optimize marker contrast as seen by both sets of cameras under ambient room lighting conditions.

The four outer corners of the mounted Aruco markers were used as fiducial locations. These points were registered to four virtual fiducials placed onto a transparent virtual tab, which was attached to the bottom of each ultrasound image read into the system. The registration alignment was calibrated by altering the location of the virtual fiducials on the virtual table. Calibration was conducted to determine optimal virtual fiducial marker placements for minimal TRE using a tissue phantom imbedded with liquid fluorescent markers, which were visible to both the cameras and the ultrasound. Calibration phantoms were constructed, and each of these phantoms had a square well molded into the surface to a depth of 3 cm. Each well was filled with a 300 nM solution of ICG in DMSO.

The ultrasound transducer was then fixed parallel to the horizontal plane (0°) with the Aruco markers facing up. The transducer and tissue phantom were in view from the fluorescence imaging cameras. The fluorescent dye in the phantom was excited using a 1 mW/cm^2^ excitation light, and the fluorescence and ultrasound images were registered. Alignment was achieved by monitoring the registration and adjusting the virtual fiducial locations until the fluorescent wells overlaid precisely with the well locations as seen by the ultrasound.

Multiple additional tissue phantoms (*n* = 6) were created to test the fidelity of the registration. Each of these phantoms were created with a single square well molded into the surface at a depth of 3 cm, and filled with a 300 nM solution of ICG in DMSO, and the fluorescence was excited at 1 mW/cm^2^ excitation light intensity. The TRE was evaluated based on the differences in edge location of the square hollow seen in the ultrasound images versus the square fluorescence imaged on the tissue phantom. Error was evaluated based on differences in translation (mm), rotation (°) and scale (% size difference) between the co-registered ultrasound and fluorescence images. To obtain more precise measurement locations, the square in each image was outlined with a best fit square contour [59]. The FRE was evaluated by drawing the locations of the virtual fiducials onto the optical image and comparing their locations with the outer registration points on the Aruco markers.

### 2.5. Multiscale Imaging with Fiber Microscope

To enable multiscale fluorescence imaging, the wide-field stereoscopic fluorescence imaging system was supplemented with an in vivo microscopic fluorescence imaging module. The microscope was built in-house, incorporating a high-resolution silicon fiber imaging bundle (IB10001000CTD Schott, Southbridge, MA, USA) as the imaging probe, to deliver excitation and collect emission light from a fluorescent target. The device utilized a 780 nm LED emitter chip connected to a variable output driver to provide fluorescence excitation to the target (M780LP1 and LEDD1B ThorLabs, Newton, NJ, USA). A dichroic mirror (69-883 Edmund Optics, Barrington, NJ, USA) was implemented to reflect the excitation light towards a focusing objective lens that directed the light onto the proximal end of the fiber imaging bundle, which then delivered the light to the target. The fiber bundle also acted to collect the subsequent fluorescence emissions, passing the collected light to the magnifying objective (MRH00041 Nikon, Tokyo, Japan). Following the objective lens, emission wavelength light was passed through the dichroic mirror, while reflecting away any returned excitation wavelength light. Passed emission light was then focused onto a filtered CCD camera. The microscopic imagery was viewed in picture-in-picture mode on the AR display.

Testing of the microscopic module included fluorescence sensitivity and resolution. Fluorescence sensitivity was determined by imaging series of ICG-labeled tissue phantoms. For microscopic imaging, only the 2 mL phantoms were imaged, since the effective imaging area of the system was limited to a 1 mm diameter circle. Concentrations of ICG used for imaging included: 50, 62.5, 100, 200, 300, 500, 1000, and 2000 nM, where the 50 nM solution corresponded to the background level. Intensity readings from the microscope were used to calculate the Signal-to-Background Ratio (SBR) at each dye concentration, and the minimum dye concentrations required to achieve an SBR of 2 was calculated. The excitation intensity for the microscope was set at 2 mW/cm^2^, measured at the microscopic objective, to achieve optimal SBRs of fluorescent data.

In addition, the resolution of the microscope was determined by imaging a USAF 1951 Resolution Target (R3L3S1P ThorLabs, Newton, NJ, USA). Resolution was determined by selecting the smallest pattern on the target in which three distinct black bars could be visualized with a contrast of 20%.

### 2.6. 3D Object Registration and AR Overlay Using Optical Markers

Projection of 3D data (i.e., volumetric renderings of MRI/CT) onto the 2D stereoscopic images recorded from the systems color or NIR stereo cameras was conducted in real time, using Aruco markers as fiducial targets [36]. Markers were placed in a quadrilateral array around the real-world target of the registration operation. Real-time detection of the markers was realized through the OpenCV Aruco library, which worked to identify the markers in each frame using the prescribed algorithm and returned their corner locations [36,60]. Registration of the projection matrix was conducted using the OpenCV libraries to solve the Perspective-n-Point (PnP) problem and find the correct 3D object pose [61,62], aligning the detected fiducial marker points with corresponding points on the projection matrix, which serve as virtual fiducials for the 3D object. In order to achieve correct pose, the registration matrices, including translation, scale and rotation, between the virtual and fiducial points must be calculated. The correct 3D registration matrix was found using OpenCV functions to estimate the appropriate affine transform between the two 3D data sets [63]. Once the registration matrix was known, the projection matrix could be registered to the stereo scene. The 3D object was then loaded into the projection matrix using the Open Graphics Libraries (OpenGL) [64], which was then used to construct a view matrix, as demonstrated by Mulligan [62]. Viewing of the co-registered 2D camera images and 3D object was also enabled via OpenGL. The 2D camera images were drawn on a frame-by-frame basis as an OpenGL background texture. Meanwhile, the 3D object was drawn onto the scene within the registered projection matrix.

Registration accuracy was assessed by co-registering rigid bodies with distinct edges, which were fully visible in both the virtual and physical world. To this end, a virtual 3D cube was created using Blender and registered to a real 3D-printed cube, which was imaged through the color cameras. The virtual cube was constructed to the same dimensions of the real cube, 50 mm^3^. Virtual registration points were set at 50 mm in a diagonal line from each of the virtual cube’s bottom corner points, and Aruco markers were placed at similar intervals from the real cube. The co-registered 3D projection and 2D camera frames were analyzed for target registration error (TRE) in terms of translation, rotation, and scale. Misalignment was calculated by comparing the locations of the co-registered cube corners. Additionally, fiducial registration error (FRE) was calculated by drawing the both locations of the virtual fiducials and their co-registered Aruco corners onto the scene, and comparing the coordinates’ locations. The physical distance between registration points was estimated using the Aruco markers, whose dimensions are known.

In order to correct for placement error, additional internal fiducial points were incorporated. Like the previous fiducials, this point was indicated using an Aruco marker. The fifth Aruco marker was affixed to a digital stylus pen. The vector from the tip of the pen to the Aruco marker was rigid, therefore the tip’s location could be determined and tracked when the marker was detected. The stylus also communicated with the PC via wireless IR link. When a button on the stylus was pressed, the current location of marker tip, as seen through the wide-field cameras, was set as a fiducial location. New fiducial registration points were created by first pointing to, then clicking on the location of the misaligned virtual anatomy. Next, the user clicked on the physical location where the previously selected anatomy should be located. The distance between the first and second selected points was calculated in *x*–*y* pixel coordinates, and this displacement was applied to the location of the originally detected fiducial marker coordinates. Effectively, this allowed the user to shift the registration in the *x*–*y* plane. Additional corrections could be applied by repeating the process. Poorly placed or unwanted fiducial selections could be removed via keystroke. Object registration in the *Z*-plane (i.e., height/depth) was also adjustable using OpenGL commands, by pressing the up or down arrows on the keyboard to effectively translate the background imaging plane to a closer or further imaging depth, respectively.

Two classes of registration error were quantified, including Fiducial Registration Error (FRE) and Target Registration Error (TRE). Virtual fiducial points, corresponding to 3D virtual object corners, were registered to and drawn on the imaged Aruco marker corners. The error between the target fiducial marker corners and their corresponding virtual fiducial points in the x and *y* axes of the imaging plane were averaged over multiple trials and reported. Additionally, TRE was measured between the corners of a 3D-printed box as seen in the imaging plane of the color cameras and the corners of a virtual box registered to the fiducial markers.

The accuracy of the detailed registration method is dependent on correct user placement of the fiducial markers. Determining correct marker placement for a particular procedure required calibration. Calibration of fiducial marker placement involved placing the markers at key reference points for the anatomy being imaged. Testing the system on a non-geometric target was conducted, using a 3D heart model as an example [65]. Fiducial markers were placed at strategic locations around a tissue phantom simulating a heart. Marker locations corresponded to the approximate locations of the edge of the patient’s aortic arch, apex, and right and left atria. Prior to imaging, a 3D heart model was uploaded into the virtual space. The 3D heart was placed within the projection matrix so that the virtual aortic arch, apex, and right and left atria would align with the virtual fiducial markers. Following registration, FRE was assessed in a similar method as described with the two cubes. Additionally, TRE was checked by placing plastic centrifuge tubes filled with a 100 nM solution of ICG in DMSO at physical locations corresponding to the simulated patient’s left common carotid artery and left ventricle. The diameter of the tube was measured in the images, and the center line was located. The center of the artery was similarly located and used as a reference point for assessing registration alignment. The described stylus was implemented here to correct for misalignments.

Temporal and spatial accuracies of the registration techniques were evaluated. Frame rates were calculated as the number of completed loops per second during the operation of each object registration mode. Time latency between when all four fiducial markers were detected and the subsequent registration was also calculated. For the lite registration method, latency between keystroke and new registration was also calculated.

The Aruco markers were detected using the color CMOS imaging sensors for testing purposes, and the fluorescence images, when present, were co-registered. Object registration began only when both CMOS sensors detected all four of the placed fiducial markers. Following initial registration, only 2 Aruco markers needed to be visible in each of the stereo camera’s captured frames for registration to continue. When one or more of the markers were no longer visible, the last known coordinate for that marker was used for registration.

## 3. Results

The system offers multimodal 3D optical imaging and AR in real time. Stereoscopic fluorescence imaging and stereoscopic color imaging can be enabled concurrently. The wearable 3D visualization is similar to the user experience of a surgical loupe, with a minimal learning curve for a surgeon.

**Figure 2:**
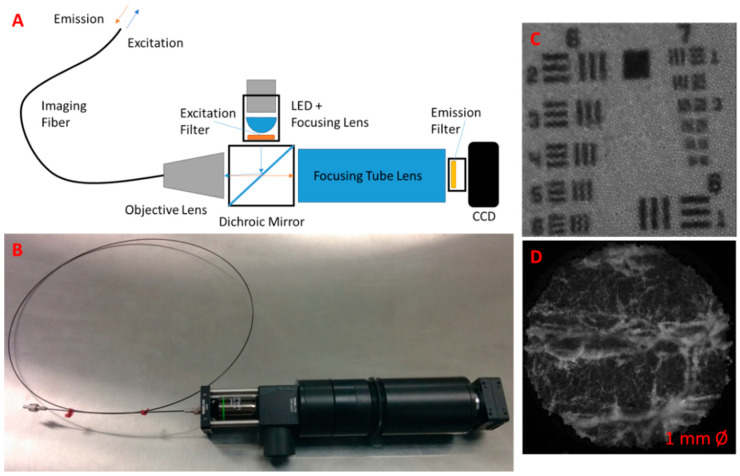
Fiber-optic fluorescence microscope for multiscale functional imaging. (**A**) Schematics of optical instrumentation, (**B**) photo of fiber optic fluorescence microscope prototype; (**C**) USAF resolution target. The system achieves fine resolution down to approximately a smallest dimension of 17.54 µm; and (**D**) the microscope can image intricate fluorescent tissue morphology.

Multiscale functional imaging was enabled by developing and integrating an in vivo fiber microscope (Figure 2). The wide-field stereoscopic fluorescence imaging can be supplemented with microscopic fluorescence imaging. The prototype passes excitation wavelength light to and from the target via a flexible fiber-optic imaging bundle (Figure 2A,B). The beam splitter separated the excitation from emission wavelengths, preventing image contamination at the imaging sensor. Additional bandpass filtration at the light source and imager further enhances sensitivity. The microscopic imagery was viewed in picture-in-picture mode on the AR display. The microscope was also tested for resolution limits using an USAF 1951 resolution target, imaged on top of the fluorescent tissue phantom so as to make the pattern visible through the microscope filters. A minimum resolvable pattern on the target was found to occur at Group 4 Element 6, which had a smallest dimension of 17.54 µm. (Figure 2C). The diameter of the microscopic image field of view is 1 mm (Figure 2D).

**Figure 3:**
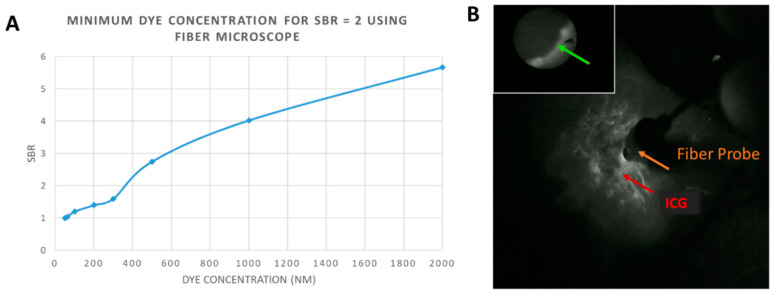
Multiscale functional imaging. (**A**) Plot of Signal-to-Background Ration versus Dye Concentration for the fiber optic microscope, when detecting indocyanine green (ICG) in a tissue phantom. The background of the phantom was set at a 50 nM ICG concentration; (**B**) Single frame from the wide-field fluorescence imaging cameras with the fiber microscopic imaging displayed in the top left corner. A microscopic fluorescent boundary not resolved by the wide-field cameras is indicated by the green arrow. The red arrow indicates ICG fluorescence in the NIR spectrum, and the fiber optic probe tip can be visualized in use.

The fiber optic microscope was tested for fluorescence sensitivity using the same type of well-patterned tissue phantoms containing varying concentrations of ICG. The minimum concentration of ICG required to achieve an SBR of at least 2 was tabulated at about 370 nM. A plot of SBR versus dye concentration was graphed (Figure 3A). The microscope was operated simultaneously with the multimodal imaging and AR display modules, displaying the microscopic image in picture-in-picture mode (Figure 3B). No noticeable latency was observed between microscopic image update and wide-field camera capture and subsequent display.

**Figure 4:**
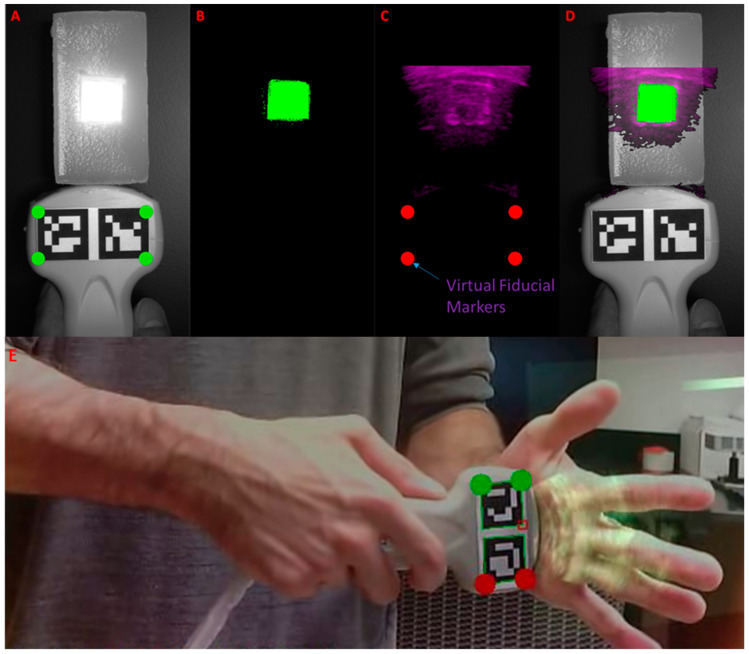
Real-time multimodal imaging with ultrasound-to-optical image registration. (**A**) Reflectance image of the phantom with detection of fiducial markers (Aruco): the virtual fiducials for registration in the array are marked in green; (**B**) Fluorescence image of phantom: fluorescence is pseudocolored in green; (**C**) B-mode ultrasound image: the cropped and color-coded ultrasound image is labeled with virtual fiducial markers in red. The fluid-filled hollow in the ultrasound image aligns with the fluorescence image; (**D**) Co-registered ultrasound/fluorescence/reflectance image; (**E**) Co-registered ultrasound/optical image of human hand.

Ultrasound imaging was integrated into the system, and ultrasound images were successfully registered to optical images using an optical marker array affixed to the ultrasound transducer (Figure 4). In the accuracy evaluating tissue phantom, fluorescence and reflectance images are registered to the b-mode ultrasound images in real time (Figure 4A–D). Furthermore, the system was used to image a human’s hand, and ultrasound images were successfully registered to optical images (Figure 4E).

The mean errors of registration were calculated for fiducial and target registration between the ultrasound and fluorescence images. The mean Fiducial Registration Error (FRE) and Target Registration Error (TRE) values are tabulated in Table 1. Target registration error was calculated between the corners of the best-fit boxes applied to the square fluorescent inclusion as it appeared in each of the co-registered images. The system achieved a mean of FRE and TRE of less than 2 mm, which is within the clinically acceptable range.

**Figure 5:**
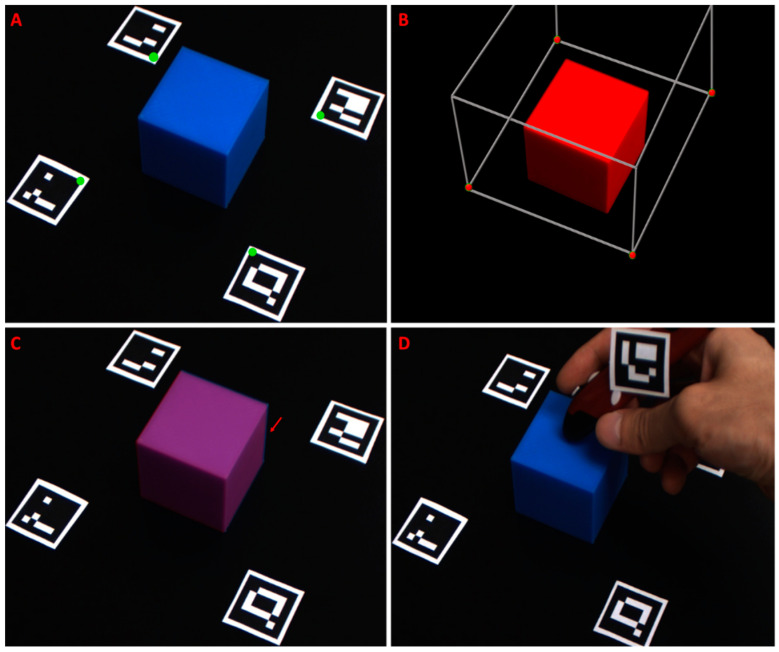
Registration accuracy assessment test for registering a 3D object with the system image sensors. (**A**) The 3D printed cube (blue) with fiducial markers placed for optimal alignment. The markers have been detected by the system, and green dots have been placed at the selected registration corners; (**B**) The virtual cube (red) in 3D space. The cube is contained within the projection matrix (gray box) whose virtual fiducial points are indicated in red; (**C**) Co-registered image of the imaged blue cube with the virtual red cube (purple). The red arrow indicates a small registration misalignment where the blue of the real-world cube can be seen; (**D**) Using the stylus with affixed fifth Aruco marker to assign a fifth interior fiducial point.

Projection of 3D data (i.e., volumetric renderings of MRI/CT) onto the 2D stereoscopic images recorded from the systems color or NIR stereo cameras was conducted in real time. Markers were placed in a quadrilateral array around the real-world target of the registration operation. The markers were identified in each frame, and their corner locations were computed. The inner upper corner of each marker was selected as a fiducial point (Figure 5A). These points were used to anchor the projection matrix, a cubic 3D volume in virtual space containing the volumetric rendering to be projected onto the 2D image (Figure 5B). Registration accuracy was assessed by co-registering rigid bodies with distinct edges, which were fully visible in both the virtual and physical world. A virtual 3D cube was created and registered to a real 3D-printed cube, which was imaged through the color cameras (Figure 5A–C). The virtual cube was constructed to the same dimensions as the real cube, 50 mm^3^.

In order to correct for placement error, additional internal fiducial points were incorporated. The fifth Aruco marker was affixed to a digital stylus pen (Figure 5D). The vector from the tip of the pen to the Aruco marker was rigid; therefore, the tip’s location could be determined and tracked when the marker was detected. The stylus also communicated with the PC via wireless IR link. When a button on the stylus was pressed, the current location of the marker tip, as seen through the wide-field color cameras, was set as a fiducial location. New fiducial registration points were created by first pointing to, then clicking on the location of the misaligned virtual anatomy. Next, the user clicked on the physical location where the previously selected anatomy should be located. The distance between the first and second selected points was calculated, and this displacement was applied to the location of the originally detected fiducial marker coordinates. Effectively, this allowed the user to shift the registration. Additional corrections could be applied by repeating the process.

Registration accuracy was assessed, and both Fiducial Registration Error (FRE) and Target Registration Error (TRE) were quantified. The mean TRE was less than 2 mm, which was within the clinically acceptable range.

The latency of the system between marker detection and object registration was less than 0.1 s. Frame rate during object registration is between 15 and 22 fps, with the current computation platform.

**Figure 6:**
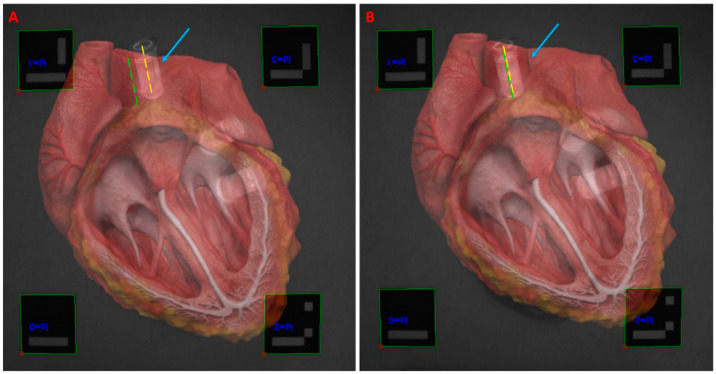
Registration of 3D heart model to fluorescent tissue phantom via fiducial markers as detected by the system cameras. (**A**) The initial registration experienced some initial misalignment (blue arrow) between the fluorescent target (yellow line) and the left common carotid artery (green line); (**B**) The misalignment was corrected using the stylus to select the misaligned points, causing the registration to shift and the target anatomies to become more closely aligned.

Testing was conducted of the object registration using both color and fluorescence image sensors as well as a practical object. The 3D heart model was registered to a tissue phantom heart with incorporated fluorescent markers (Figure 6). During the procedure, the fifth marker affixed to the handheld stylus was implemented to correct for registration error. The latency between the fifth fiducial marker assignment and registration update was less than 0.1 s. The FRE measured during the procedure averaged 1.1 mm, consistent with the findings from Table 2. The mean TRE, measured between the center lines of the fluorescence target and the center lines of the virtual blood vessels to which they should register, was 1.8 mm.

## 4. Discussion

In this paper, we have constructed a novel multimodal, multiscale imaging system with augmented reality capability. Multimodal stereoscopic imaging and 3D display were enabled. The multiscale fluorescence imaging facilitated assessing the functional status of tissues, extending the minimal resolution of fluorescence imaging to ~17.5 µm. The fiber optic microscope proved effective in identifying fine fluorescent details. The picture-in-picture presentation of multiscale fluorescence imaging data was easy to use and intuitive. The utility of the device may be further improved through the addition of multiple magnification lenses. Adding lensing to the distal tip of the fiber imaging bundle could improve function by increasing the field of view.

The ability to have multiscale fluorescence imaging and functional microscopy has the potential to provide better intraoperative image guidance [66,67]. Surgical setting often needs different scale to accommodate various imaging application, such as tissue perfusion test and tumor margin assessment [12,13,68]. Fluorescence imaging at different scales hold great promise for solving many clinical problems [69].

The use of printed fiducial markers as registration points for ultrasound and MRI imaging proved convenient, cost-effective, and accurate. TRE of less than 2 mm is consistent with a clinically acceptable range of registration accuracy. Ultrasound and fluorescence imaging provides complementary information. Ultrasound imaging provides structural information, low contrast, and deeper tissue penetration; fluorescence imaging provides functional information, high contrast, and deeper tissue penetration. MRI-based 3D model registration can become useful for surgical navigation and surgical training. Low-cost surgical navigation and 3D AR visualization have the potential to be applied to medical education and training. The marker-stylus correction was useful and practical for improving registration accuracy. The low latency and high frame rate of the prototype system have shown the promise of applying the reported techniques in clinically relevant settings in the future.

The multimodal, multiscale imaging system with AR capability can potentially be applied to multiple surgical subspecialities, such as general surgery, surgical oncology, neurosurgery, plastic surgery, and orthopedic surgery. Multimodal image guidance during surgery can provide surgeons with complementary information of structural imaging and functional imaging [70,71,72,73]. Strengths of each modality can be combined, and shortcomings of individual can be avoided. AR visualization has been shown to facilitate surgical planning, intraoperative imaging, and treatment monitoring [6,68,74].

### Limitations and Future Work

The current study focuses on system development, feasibility study, and accuracy characterization in a benchtop setting. In the future, we will investigate the effectiveness of the system in animal, cadaveric, and human studies. We will further investigate the application of the system in surgical training and medical training. The current study focuses on b-mode ultrasound, and we will investigate the registration of 3D ultrasound. In the future, we also plan to investigate MRI-based 3D model registration in patients.

## 5. Conclusions

Multimodal imaging leverages the strengths of each integrated method to offset the weaknesses. This paper introduces a novel head-mounted AR-capable fluorescence imaging system incorporating microscopy, ultrasound, and 3D tomographical imaging display. The system is tested for image registration accuracy, temporal performance, and fiber optic sensitivity. Multimodal, multiscale imaging with AR capability represents a promising platform for image-guided surgery.

## Figures and Tables

**Table 1:** Error measurements for ultrasound to fluorescence image registration using Aruco fiducial markers.

	FRE		TRE			
	Tx (mm)	Ty (mm)	Tx (mm)	Ty (mm)	R (^o^)	S (%)
Mean	1.22	1.15	1.86	1.81	2.19	3.89
STD	0.35	0.30	0.53	0.55	1.42	2.07
Maximum	1.8	1.8	2.50	2.55	6.00	7.50
Minimum	0.7	0.8	0.80	0.80	0.50	1.10

Measurements include translational error in the *x*-direction (Tx) and in the *y*-axis (Ty), as well as rotational error (R) and scaling error (S). Both the Fiducial Registration Error (FRE) and Target Registration Error (TRE) were determined.

**Table 2:** Error measurements for 3D object registration using Aruco fiducial markers.

	FRE		TRE			
	Tx (mm)	Ty (mm)	Tx (mm)	Ty (mm)	R (^o^)	S (%)
Mean	1.41	1.11	1.74	1.37	0.95	1.01
STD	0.31	0.37	0.47	0.36	0.63	0.00
Maximum	1.9	1.8	2.4	2	2.5	1.8
Minimum	0.8	0.5	0.8	0.8	0.1	0.4

Measurements include translational error in the *x*-direction (Tx) and in the *y*-axis (Ty), as well as rotational error (R) and scaling error (S). Both the Fiducial Registration Error (FRE) and Target Registration Error (TRE) were determined.

## Data Availability

Data available from authors on request.
